# When do anatomical priors help? Missing-modality robustness in small-sample dual-modal brain MRI region segmentation

**DOI:** 10.3389/fneur.2026.1908175

**Published:** 2026-09-09

**Authors:** Yuyan Zhang

**Affiliations:** School of Computer and Communication Engineering, University of Science and Technology Beijing, Beijing, China

**Keywords:** anatomical prior, brain MRI segmentation, medical image analysis, missing modality, multimodal MRI, probabilistic atlas, robust segmentation, U-Net

## Abstract

Anatomical priors are often assumed to improve medical image segmentation, but their role can be ambiguous when strong image evidence is available. We study this question in a controlled small-sample dual-modal brain MRI setting with 13-class coarse brain-region segmentation from T1 and FLAIR images. Using a quality-controlled 78-case subset and a subject-disjoint five-fold protocol, we compare compact U-Net variants, coordinate channels, modality dropout, fold-wise index-space atlas conditioning, and a full-to-missing modality consistency variant, FW-AtlasMC. Fold-wise probabilistic spatial priors are built only from training labels, and an atlas-only baseline quantifies spatial prior strength without model training. Under full T1+FLAIR input, learned methods are tightly clustered in Dice, and atlas-informed models do not significantly improve full-modality Dice over a 2.5D U-Net baseline. However, among learned image-conditioned models under missing-modality inference, FW-AtlasMC improves FLAIR-only Dice and reduces average performance drop compared with 2.5D U-Net, modality dropout, and FW-Atlas. FW-AtlasMC combines atlas-informed inputs with missing-modality training and full-to-missing consistency. Validation-only sensitivity analysis further indicates that the chosen consistency weight represents a reasonable trade-off within the tested range. These results suggest that anatomical priors are better understood as structural stabilizers under degraded modality information, rather than as universal full-modality accuracy boosters.

## 1 Introduction

Multimodal brain MRI segmentation benefits from complementary tissue contrast; however, practical imaging protocols often face incomplete or unreliable modalities. In small datasets, this problem is amplified: A model may perform well when all modalities are present but collapse when one contrast is missing. Anatomical priors, such as probabilistic atlases, offer an appealing form of structural regularization because many brain regions have stable spatial organization. However, it is not always clear whether such priors improve segmentation accuracy when complete image information is already available, or whether they mainly help when image evidence is degraded.

We investigate this distinction in a 13-class brain-region segmentation task using T1 and FLAIR MRI. The task is coarse anatomical region segmentation rather than lesion detection. This setting is useful because anatomical regularity is strong, the sample size is modest, and missing-modality behavior can be tested directly under full T1+FLAIR, T1-only, and FLAIR-only inference. Our central question is: when do fold-wise anatomical priors help?

This study uses a controlled comparison of compact baselines under a unified split, evaluator, and metric policy to isolate the role of fold-wise anatomical priors. The key finding is nuanced. Under full T1+FLAIR input, atlas-informed models do not significantly improve Dice over a 2.5D U-Net baseline. In contrast, FW-AtlasMC, which combines atlas-informed inputs with full-to-missing consistency, substantially improves missing-modality robustness among learned image-conditioned models, especially under FLAIR-only inference.

Our contributions are as follows:

1. We provide a controlled benchmark for 13-class small-sample dual-modal brain-region segmentation using a 78-case quality-controlled subset and a unified five-fold protocol.
2. We construct fold-wise index-space probabilistic spatial priors from training labels only and include an atlas-only baseline to quantify spatial prior strength.
3. We compare compact U-Net variants, coordinate priors, modality dropout, FW-Atlas, and FW-AtlasMC under full T1+FLAIR, T1-only, and FLAIR-only inference.
4. We show that atlas-informed models do not significantly improve full-modality Dice, but FW-AtlasMC improves FLAIR-only Dice and reduces average missing-modality performance drop among learned image-conditioned models.

## 2 Related work

### 2.1 Medical image segmentation baselines

U-Net remains a standard architecture for biomedical segmentation because it combines local detail with multi-scale context (1). 3D U-Net and DeepMedic extended convolutional segmentation to volumetric medical data (2, 3). nnU-Net further showed that careful self-configuration can produce strong and robust medical segmentation baselines across many tasks (4). In this study, nnU-Net is used to contextualize the work, while the quantitative analysis is restricted to compact baselines matched within the present protocol.

### 2.2 Anatomical and atlas priors

Atlas-based and probabilistic spatial priors have a long history in brain MRI analysis, including Bayesian and deformable segmentation formulations (5–7). Deep networks can also incorporate spatial or anatomical prior information as auxiliary inputs or regularizers. Building on this established role of atlas priors, we examine whether a fold-wise atlas built only from training labels functions primarily as an accuracy booster or as a robustness stabilizer.

### 2.3 Missing-modality segmentation

Missing MRI contrasts have been addressed through modality-specific statistics, latent completion, feature fusion, compensation, and modality dropout. HeMIS aggregates modality-specific features (8), while HVED jointly models completion and segmentation (9). ModDrop and ModDrop++ train networks to tolerate absent inputs (10, 11). Recent brain-tumor methods include RFNet, ACN, mmFormer, MFI, SMU-Net, and MGML (12–17); these primarily target arbitrary missing combinations through region-aware fusion, adversarial co-training, transformers, feature interaction, style matching, or meta-guidance. Our setting differs: We study small-sample dual-modal coarse brain-region segmentation and ask whether a leakage-controlled fold-wise spatial prior is a full-modality accuracy booster or a missing-modality stabilizer.

### 2.4 2.5D and anisotropic segmentation

Anisotropic volumes often motivate 2D or 2.5D segmentation strategies that use neighboring slices without fully 3D convolutional cost (18, 19). Evaluation also requires care because metric choice can change conclusions (20). Our baselines include 2D U-Net and 2.5D U-Net variants so that atlas conditioning is compared against compact models that already exploit local through-plane context.

## 3 Methods

### 3.1 Dataset and task

We use the CBCS-MCI/brainlobe analysis subset for 13-class brain-region segmentation from T1 and FLAIR MRI. The analysis includes 78 quality-controlled cases derived from 80 complete cases, with T1, FLAIR, and label volumes on a common 512 × 512 × 16 grid. Two cases with verified metadata-level image-label misalignment were excluded before model development and evaluation. All methods use the same five-fold protocol with subject-disjoint train, validation, and test partitions; validation is used only for checkpoint selection and hyperparameter choice, and the test split is used only for final evaluation.

The CBCS-MCI study was approved by the ethics committees of all participating centers (approval No. YN2023-011-02) and registered in the Chinese Clinical Trial Registry (ChiCTR2500107979) (21). All images used in this analysis were de-identified, and no patient-identifying information is reported. The de-identified brainlobe subset was used for secondary methodological analysis under the approved CBCS-MCI study framework and dataset-use constraints. Segmentation masks were dataset-provided brainlobe annotations; this study did not relabel images or modify the annotation protocol. Code and analysis outputs may be made available subject to dataset redistribution constraints.

The current analysis metadata did not include scanner model, acquisition site, or detailed protocol fields; accordingly, scanner- or protocol-level subgroup effects were outside the scope of this analysis. Table 1 summarizes missing-modality training and inference handling.

**Table 1:** Missing-modality training and inference treatment.

Method	Training input treatment	Missing-modality inference
Atlas-only	No training; fold-wise atlas argmax	Modality-independent
2D U-Net	Full T1+FLAIR only	Absent modality zeroed
2.5D U-Net	Full T1+FLAIR stack only	Absent modality zeroed
2.5D + Coord	Full images plus coordinate channels	Absent image modality zeroed; coordinates unchanged
2.5D + ModDrop	Random modality zeroing during training	Absent modality zeroed
FW-Atlas	Full images plus atlas confidence/entropy	Absent image modality zeroed; atlas unchanged
FW-AtlasMC	Full/T1-only/FLAIR-only supervised branches plus KL consistency	Same zeroing protocol; atlas unchanged

Zeroing is applied in normalized tensor space for image channels only.

### 3.2 Baselines and atlas construction

We compare seven methods: atlas-only, 2D U-Net, 2.5D U-Net, 2.5D U-Net with coordinate channels, 2.5D U-Net with modality dropout, FW-Atlas, and FW-AtlasMC. All learned models are compact U-Net variants with comparable parameter counts.

All clean cases share a common matrix size of 512 × 512 × 16. Quality control verified the T1/FLAIR/label triplet matrix size, spacing, origin, and direction metadata for each case; confirmed metadata-level image-label misalignment was excluded before model development. After excluding the two metadata-misaligned cases, T1, FLAIR, and label maps are consistent within each case in size, spacing, origin, and direction. In-plane spacing is nearly constant across subjects (0.46875–0.46880 mm), whereas through-plane spacing varies from 6.00 to 9.10 mm (median 8.40 mm). We therefore construct the atlas in the common voxel-index grid and refer to it as a fold-wise index-space anatomical prior, distinct from a deformably registered physical-space population atlas.

For fold *k*, let T_*k*_ be the training cases. The class probability prior is defined in Equation 1 as


$$\begin{array}{l} A_{c}\left(\upsilon \right)=\frac{1}{\left|T_{k}\right|}\underset{i\in T_{k}}{\sum }1\left[Y_{i}\left(\upsilon \right)=c\right], \end{array}$$ (1)

where *v* indexes a voxel in the common grid. The atlas-only baseline predicts $\arg\underset{c}{\max}A_{c}\left(\upsilon \right)$ at each voxel. FW-Atlas and FW-AtlasMC use two atlas-derived channels at the center slice: confidence $C\left(\upsilon \right)=\underset{c}{\max}A_{c}\left(\upsilon \right)$ and entropy $H\left(\upsilon \right)=-\underset{c}{\sum }A_{c}\left(\upsilon \right)\log\left(A_{c}\left(\upsilon \right)+\epsilon \right)$. These two channels summarize the fold-wise class-probability atlas as compact certainty and uncertainty maps. Using two atlas-derived channels avoids feeding all 13 class-probability maps, limiting overhead and reducing over-reliance on label-derived priors. The class-probability atlas is stored in (*C, Z, Y, X*) order before deriving confidence and entropy maps.

### 3.3 FW-Atlas and FW-AtlasMC

The 2.5D input uses five adjacent slices per modality, (*z*−2, …, *z*+2), with replicate padding at volume boundaries. The model predicts the center-slice label. Thus, the plain 2.5D model has 10 image channels. The coordinate baseline adds normalized *x*, *y*, and *z* coordinate channels, for 13 total input channels. FW-Atlas and FW-AtlasMC add atlas confidence and entropy channels, for 12 total input channels. Preprocessing for model input was limited to per-case non-zero-region intensity normalization, 2.5D slice stacking with replicate boundary padding, and channel zeroing for simulated missing-modality inference; no deformable inter-subject registration or data augmentation was used.

FW-Atlas augments the T1/FLAIR slice-stack input with the two fold-wise atlas-derived prior channels. FW-AtlasMC extends this input design with full-to-missing consistency under three inference modes: full T1+FLAIR, T1-only with FLAIR channels zeroed, and FLAIR-only with T1 channels zeroed. Zeroing is applied in the normalized tensor space. Atlas and coordinate channels remain unchanged when an image modality is missing. Its loss is defined in Equation 2 as:


$$\begin{array}{l} L=L_{DC}\left(P_{f},Y\right)+0.5L_{DC}\left(P_{T1},Y\right)+0.5L_{DC}\left(P_{F},Y\right)\\ \;+\lambda \left[D_{KL}\left(P_{T1}∥\text{sg}\left(P_{f}\right)\right)+D_{KL}\left(P_{F}∥\text{sg}\left(P_{f}\right)\right)\right], \end{array}$$ (2)

where L_*DC*_ is Dice plus cross entropy, *P*_*f*_ is the full-modality prediction, *P*_*T*1_ and *P*_*F*_ are missing-modality predictions, and sg denotes stop-gradient. Cross entropy includes background, whereas the Dice term excludes background. We use λ = 0.1 as a conservative consistency weight, keeping KL regularization secondary to the supervised Dice–cross-entropy losses.

All learned models use AdamW with learning rate 10^−3^, weight decay 10^−4^, maximum 200 epochs, and patience 50. Training batch size is 4 for 2D U-Net and 2 for all other learned models. The base random seed is 2026, and the fold-specific seed is 2026+*k*. No data augmentation was used during training. The checkpoint metric is foreground validation mean Dice computed on reconstructed 3D validation volumes. Best checkpoints are selected using validation data only.

**Figure 1:**
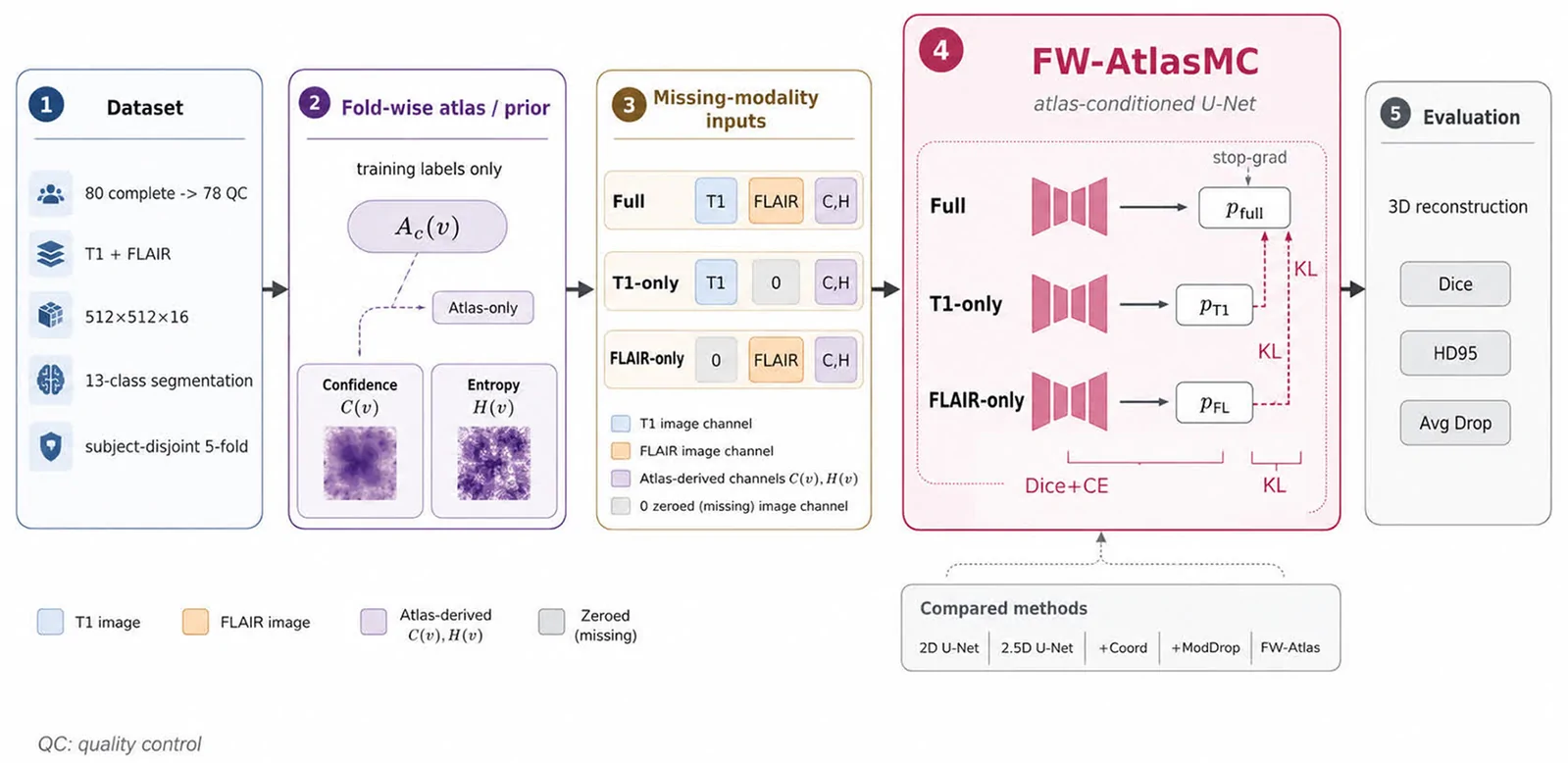
Study and methodological overview. Quality-controlled dual-modal MRI cases (T1 and FLAIR) were segmented into 13 classes under a five-fold subject-disjoint protocol. For each fold, the anatomical atlas [*A*_*c*_(υ)] was constructed from training labels only and summarized as confidence [*C*(υ)] and entropy [*H*(υ)] atlas-derived channels; the atlas-only baseline used the class-probability atlas directly. Missing-modality inference was simulated by zeroing the corresponding normalized image-modality channels while retaining atlas-derived channels. FW-AtlasMC uses full-, T1-only-, and FLAIR-only branches with supervised Dice/CE losses and KL consistency from missing-modality predictions toward the stop-gradient full-modality prediction. Slice-wise predictions were reconstructed in 3D and evaluated using Dice, HD95, and average performance drop.

We assess the consistency weight with validation-only sensitivity analysis. The five values λ∈{0, 0.05, 0.1, 0.2, 0.5} are screened on fold 0, followed by five-fold validation analysis for λ∈{0, 0.1, 0.2}. At λ = 0, all three supervised Dice–cross-entropy branches are retained and only the KL consistency term is removed. Every sensitivity checkpoint is still selected exclusively by full-modality foreground validation mean Dice on reconstructed validation volumes; missing-modality scores and test data are not used for selection. Validation-only sensitivity supports λ = 0.1 as a reasonable trade-off within the tested range. The final held-out test evaluation used this originally specified setting. The overall study and methodological workflow is summarized in Figure 1.

## 4 Experiments

All reported quantitative results were derived from held-out test predictions from the five-fold evaluation. We evaluate Dice, HD95, small-class Dice, parameter count, and forward reconstruction time. Dice and HD95 are computed on reconstructed 3D volumes. Mean Dice excludes background. HD95 uses physical spacing in (*Z, Y, X*) order. Small-class Dice uses labels {5, 7, 9, 10, 11, 12}, corresponding to insula, hippocampus, brainstem, cerebellum, and corpus callosum subregions.

For missing-modality evaluation, we test full T1+FLAIR, T1-only, and FLAIR-only inference. The average drop is the mean Dice decrease from full input to T1-only and FLAIR-only settings. Statistical analysis uses case-level paired comparisons when the same test cases are available. We report bootstrap 95% confidence intervals, paired *t*-tests, Wilcoxon tests, and standardized paired effect sizes. Forward reconstruction time measures model inference plus CPU transfer during slice-wise volume reconstruction and is reported as a profiling measure rather than an end-to-end deployment runtime.

Efficiency is profiled separately from training with batch size 1 and a 512 × 512 center-slice input using each method's actual channel count. MACs include Conv2d and ConvTranspose2d operations only; FLOPs are defined as twice the MAC count. Batch normalization, activations, pooling, softmax, I/O, atlas construction and lookup, CPU transfer, and post-processing are excluded. Per-volume estimates multiply center-slice compute by 16 reconstructed slices. Because the atlas-only baseline has no trainable neural network, atlas construction, lookup, and argmax operations are excluded from neural-network MAC/FLOP estimates.

## 5 Results

### 5.1 Full-modality performance

Table 2 shows that learned methods are tightly clustered under full T1+FLAIR input, with Dice values around 0.798–0.802. The best full-modality Dice is obtained by 2.5D U-Net with modality dropout: 0.8021 ± 0.0196. FW-Atlas obtains 0.7989 ± 0.0207, and FW-AtlasMC obtains 0.7978 ± 0.0219. Same-case paired tests found no evidence of a full-modality Dice improvement for FW-Atlas or FW-AtlasMC over 2.5D U-Net. These results indicate that anatomical priors are not a universal full-modality accuracy improvement in this task.

Table 2Main quantitative results.

**Table:** 

Panel A. Full-modality segmentation performance.				
Method	Params	Dice ↑	HD95 ↓	Small dice ↑
Atlas-only	0	0.6284 ± 0.0374	12.39 ± 10.34	0.5810 ± 0.0399
2D U-Net	7.76M	0.8011 ± 0.0192	10.24 ± 9.85	0.8024 ± 0.0235
2.5D U-Net	7.77M	0.7990 ± 0.0183	10.18 ± 9.92	0.8034 ± 0.0186
2.5D + Coord	7.77M	0.8001 ± 0.0237	10.06 ± 10.14	0.8042 ± 0.0244
2.5D + ModDrop	7.77M	0.8021 ± 0.0196	9.96 ± 9.91	0.8018 ± 0.0215
FW-Atlas	7.77M	0.7989 ± 0.0207	10.21 ± 9.89	0.8032 ± 0.0231
FW-AtlasMC	7.77M	0.7978 ± 0.0219	10.26 ± 9.89	0.8004 ± 0.0237

**Table:** 

Panel B. Missing-modality robustness.				
Method	Full dice	T1-only dice	FLAIR-only dice	Avg drop ↓
2D U-Net	0.8010	0.4716	0.0337	0.5484
2.5D U-Net	0.7988	0.5394	0.0417	0.5082
2.5D + Coord	0.7997	0.6293	0.1526	0.4088
2.5D + ModDrop	0.8018	0.7968	0.0677	0.3695
FW-Atlas	0.7986	0.6936	0.1367	0.3835
FW-AtlasMC	0.7974	0.7908	0.3876	0.2082

**Table:** 

Panel C. Compact efficiency profile.						
Method	Input ch.	Params	Param overhead vs. 2.5D	MACs/slice	FLOPs/16 slices	ForwardReconTime (s)
2D U-Net	2	7.763M	−0.0297%	48.310G	1.546T	0.145
2.5D U-Net	10	7.765M	0	48.914G	1.565T	0.160
2.5D + Coord	13	7.766M	+0.0111%	49.140G	1.572T	0.187
2.5D + ModDrop	10	7.765M	0	48.914G	1.565T	0.160
FW-Atlas	12	7.766M	+576 params (+0.0074%)	49.065G	1.570T	0.170
FW-AtlasMC	12	7.766M	+576 params (+0.0074%)	49.065G	1.570T	0.168

Panel **A** reports fold-level mean ± standard deviation for full T1+FLAIR performance on the 78-case quality-controlled split. Panel **B** reports case-level mean Dice for learned image-conditioned models under full and missing-modality inference; Avg Drop is the average Dice decrease from full input to T1-only and FLAIR-only settings. Panel **C** reports batch-1 512 × 512 center-slice inference estimates; FLOPs are defined as 2 × MACs, per-volume estimates assume 16 slices, and ForwardReconTime denotes forward reconstruction time. Representative missing-modality comparisons use the same held-out case within each fold as the pairing unit and apply Holm correction across planned comparisons: FLAIR-only Dice vs. 2.5D U-Net, Δ = 0.3459, 95% CI [0.3105, 0.3810], *d*_*z*_ = 2.12, Holm-adjusted *p* < 0.001; Avg Drop vs. 2.5D U-Net, Δ = −0.3000, 95% CI [–0.3292, –0.2698], *d*_*z*_ = −2.20, Holm-adjusted *p* < 0.001. MACs count Conv2d and ConvTranspose2d only; batch normalization, activations, pooling, softmax, I/O, atlas construction/lookup/argmax, CPU transfer, and post-processing are excluded.

The atlas-only baseline reaches Dice 0.6284 ± 0.0374 and small-class Dice 0.5810 ± 0.0399 under the full evaluation setting. This is strong for a no-image predictor and confirms substantial spatial regularity. Nevertheless, all learned models outperform atlas-only under full T1+FLAIR input, showing that image-conditioned segmentation remains necessary when both modalities are available.

The efficiency panel in Table 2 shows that atlas conditioning has small architectural overhead. FW-Atlas and FW-AtlasMC add 576 parameters (0.0074%) relative to 2.5D U-Net and increase estimated per-slice MACs from 48.914G to 49.065G. Their empirical forward reconstruction time remains comparable to that of the 2.5D baseline. FLOPs and MACs are computed only for convolutional operations in the neural network. They do not include I/O, atlas construction, argmax, or post-processing. Thus, these estimates characterize model-level computational overhead.

### 5.2 Missing-modality robustness

**Figure 2:**
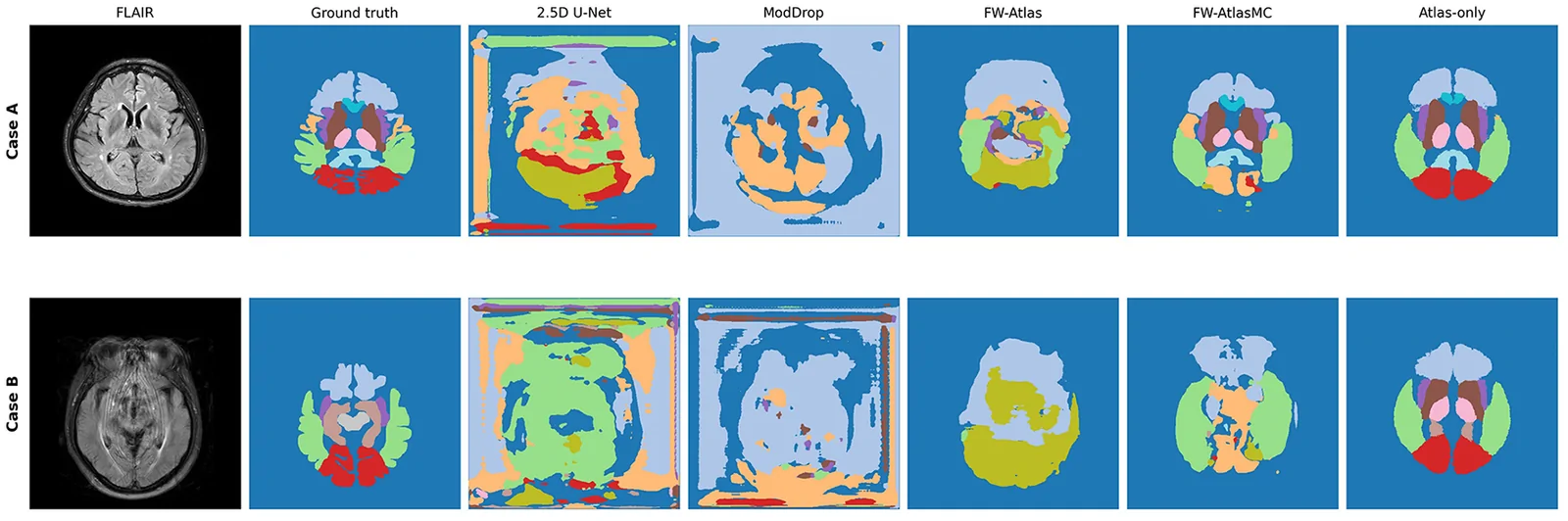
Qualitative comparison on two held-out FLAIR-only cases. All learned predictions are obtained under FLAIR-only input. Atlas-only is generated from the corresponding fold-wise training-label atlas and does not use image intensities. These examples illustrate representative FLAIR-only behavior; quantitative conclusions are based on paired statistical analyses.

Table 2 Panel B shows the main positive result among learned image-conditioned models. FW-AtlasMC has the lowest average missing-modality Dice drop, 0.2082, compared with 0.5082 for 2.5D U-Net, 0.3695 for modality dropout, and 0.3835 for FW-Atlas. Representative FLAIR-only qualitative comparisons are shown in Figure 2. The largest difference appears under FLAIR-only inference: FW-AtlasMC reaches Dice 0.3876, compared with 0.0417 for 2.5D U-Net, 0.0677 for modality dropout, and 0.1367 for FW-Atlas.

Missing-modality-specific paired tests support these differences using the same held-out case within each fold as the pairing unit. FW-AtlasMC improves FLAIR-only Dice over 2.5D U-Net (Δ = 0.3459, 95% CI [0.3105, 0.3810], *d*_*z*_ = 2.12, Holm-adjusted *p* < 0.001), modality dropout (Δ = 0.3199, 95% CI [0.2791, 0.3591], Holm-adjusted *p* < 0.001), and FW-Atlas (Δ = 0.2509, 95% CI [0.2204, 0.2820], Holm-adjusted *p* < 0.001). It also reduces Avg Drop relative to 2.5D U-Net by 0.3000 (95% CI [–0.3292, –0.2698], *d*_*z*_ = −2.20, Holm-adjusted *p* < 0.001). Wilcoxon tests give the same conclusion, and all planned FLAIR-only Dice and Avg Drop comparisons remain significant after Holm correction.

Atlas-only requires separate interpretation in the missing-modality setting. Because it is modality-independent, its prediction is unchanged when T1 or FLAIR is missing, and Avg Drop is not a meaningful robustness measure for it. Its Dice of 0.6284 is higher than the FLAIR-only Dice of FW-AtlasMC. Thus, the result is best interpreted as improved robustness among learned image-conditioned models rather than superiority over the modality-independent atlas-only reference. FW-AtlasMC is the most stable learned image-conditioned model and substantially narrows the gap between image-conditioned models and the no-image spatial prior when image evidence is severely degraded.

#### 5.2.1 Validation-only sensitivity analysis

A validation-only sensitivity analysis was conducted for the consistency weight λ. Values of λ = 0, 0.1, and 0.2 yielded similar full-modality validation Dice, indicating that λ = 0.1 is a reasonable trade-off within the tested range. The observation that λ = 0 produced stronger FLAIR-only Dice and lower Avg Drop in validation further suggests that robustness depends on the broader training design, not on the KL consistency term alone. Checkpoints were selected only by full-modality validation Dice, and the final test evaluation used the originally specified λ = 0.1 setting.

### 5.3 Per-class behavior and qualitative results

Per-class results show a heterogeneous pattern across anatomical regions. FW-AtlasMC is best for temporal lobe and hippocampus, whereas the coordinate baseline has the best aggregate small-class Dice. Brainstem and cerebellum achieve high Dice across methods, while corpus callosum subregions remain more challenging. This class-level behavior supports a cautious interpretation: Atlas-informed consistency is most useful as a robustness mechanism, not as a universal per-class improvement.

## 6 Discussion

The full-modality results clarify the operating regime of atlas conditioning. Compact U-Net baselines may already learn much of the anatomical regularity when both T1 and FLAIR are available. In that setting, an atlas prior can be redundant, and adding atlas channels does not guarantee higher Dice. This finding indicates that atlas conditioning is not sufficient to improve full-modality accuracy in this setting: FW-AtlasMC is slightly lower than FW-Atlas and modality dropout on full-modality Dice.

The missing-modality results suggest a different role for anatomical priors. When one modality is absent, appearance information becomes unreliable or incomplete. The atlas then acts as a structural fallback, and full-to-missing consistency encourages predictions under missing input to remain compatible with full-input predictions. This may help explain why FW-AtlasMC is particularly effective for FLAIR-only inference, where image-conditioned baselines show marked degradation.

The atlas-only baseline is important for interpretation. It estimates an unconditional spatial predictor *p*(*y*∣*v*), whereas FW-AtlasMC estimates an image-conditioned predictor *p*(*y*∣*x, v*). Under FLAIR-only inference, weak or ambiguous appearance cues may reduce the reliability of an image-conditioned prediction, while atlas-only remains unchanged. Consequently, FW-AtlasMC does not exceed atlas-only in this condition. Its contribution is instead to preserve full-modality image-conditioned capability while substantially reducing the degradation of learned baselines when one modality is absent.

The sensitivity analysis suggests that λ = 0.1 is a reasonable trade-off within the tested range. Robustness arises from the combination of atlas conditioning, missing-modality supervision, and consistency regularization, rather than the KL term alone.

Limitations remain. This is a single-dataset, small-sample study. The atlas used in this study is a voxel-index-space prior constructed without deformable registration. It is not a physical-space population atlas and does not directly account for anatomical variability across subjects. Individual morphology, local anatomical deformation, atrophy, and differences in slice thickness or acquisition geometry are therefore only indirectly represented through the shared voxel-index grid. Averaging in index space can also smooth variable boundaries. Under full-modality conditions, such averaged priors may become redundant or overly smooth. These properties may explain why the prior is redundant or mildly restrictive when both image contrasts provide strong evidence, limiting full-modality gains. The present evidence consequently supports the prior as a dataset-specific structural fallback under missing-modality inference, not as a universal accuracy booster. The quantitative comparison is limited to compact baselines under the present protocol; broader benchmark positioning remains outside the scope of this study. FLAIR-only Dice improves substantially with FW-AtlasMC but remains far below full-modality performance. FLOPs/MACs omit non-convolutional operations, and forward reconstruction time is reported as a profiling measure rather than a clinical deployment benchmark. Finally, labels follow the original dataset definitions, including dataset-provided class naming conventions documented separately.

## 7 Conclusion

This controlled study shows that fold-wise index-space anatomical priors do not necessarily improve full-modality Dice in small-sample dual-modal brain MRI region segmentation. However, among learned image-conditioned models, FW-AtlasMC improves FLAIR-only Dice and reduces average missing-modality performance drop. The no-image atlas-only baseline remains a strong reference when image evidence is severely degraded, emphasizing that anatomical priors should be evaluated not only as accuracy boosters but also as structural stabilizers for degraded-input segmentation.

## Data Availability

The data analyzed in this study are subject to dataset-use and redistribution constraints. Code and analysis outputs may be made available subject to these constraints. Further inquiries can be directed to the corresponding author.
